# Adding Evidence to Plasmacytoma: A Case Series

**DOI:** 10.7759/cureus.68883

**Published:** 2024-09-07

**Authors:** Rashmi Singh, Aaditya Prakash, Anamika Kumari, Sweta Sinha

**Affiliations:** 1 Radiation Oncology, Rajendra Institute of Medical Sciences (RIMS), Ranchi, IND; 2 Radiation Oncology, Tata Main Hospital, Jamshedpur, IND; 3 Radiation Oncology, Ram Janam Sulakshana Pandey (RJSP) Cancer Hospital, Ranchi, IND; 4 Oncopathology, Saar Diagnostics, Ranchi, IND

**Keywords:** extramedullary plasmacytoma, plasmacytoma, radiotherapy, response, solitary bone plasmacytoma

## Abstract

Plasmacytoma is a rare tumor of plasma cells with two primary variants: solitary bone plasmacytoma (SBP) and extramedullary plasmacytoma (EMP). It poses diagnostic challenges at times. Radiotherapy (RT) is the curative modality in the majority of cases. We share a case series with the aim of adding evidence to the literature about plasmacytoma and its clinical presentation, diagnostic challenges, and outcome with RT.

## Introduction

Plasmacytoma is a tumor with localized proliferation of plasma cells. It is quite rare, comprising 1%-2% of plasma cell neoplasms. It can affect either bone or soft tissues and so is classified as solitary bone plasmacytoma (SBP) or extramedullary plasmacytoma (EMP), respectively. Simultaneously, for a case to be of plasmacytoma, there should not be any associated other bony lesions, hypercalcemia, renal insufficiency, and anemia [1].

Due to its presentations at varied anatomical locations and with advanced imaging, it poses diagnostic challenges at times. Being extremely radiosensitive, radiotherapy (RT) has been the standard definitive treatment modality, which gives prolonged local control and even cure, especially in EMP. So, we share a case series with the aim of adding evidence to the literature about plasmacytoma and its clinical presentation, diagnostic challenges, and outcome with RT. We followed the CAse REport (CARE) reporting guidelines for writing this case series [2].

## Case presentation

Case 1

**Figure 1:**
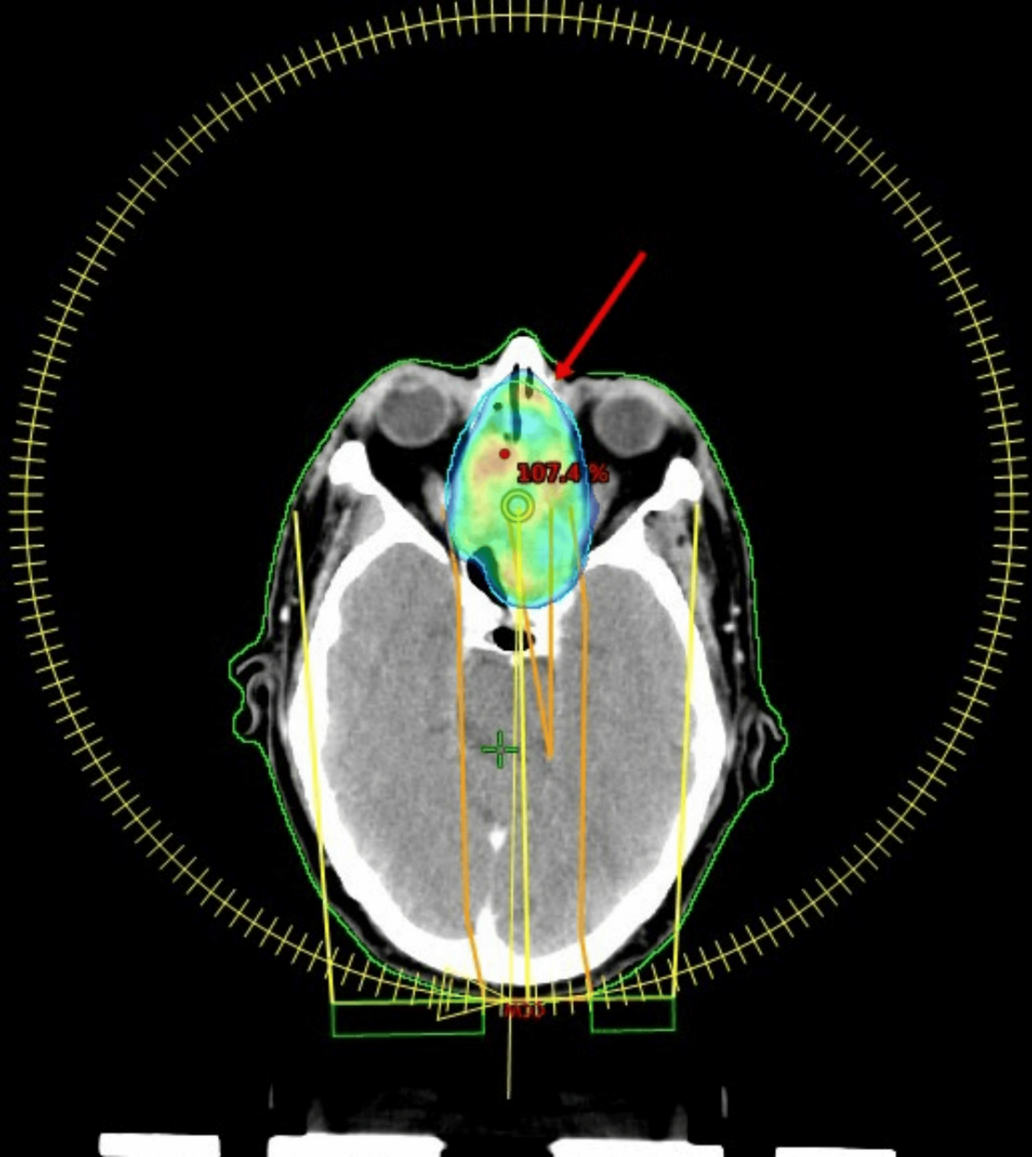
Case 1 with EMP of the nasopharynx treated with VMAT The arrow points to the PTV covering the nasopharynx, nasal cavity, and sphenoid sinus. EMP: extramedullary plasmacytoma, VMAT: volumetric modulated arc therapy, PTV: planning target volume

**Figure 2:**
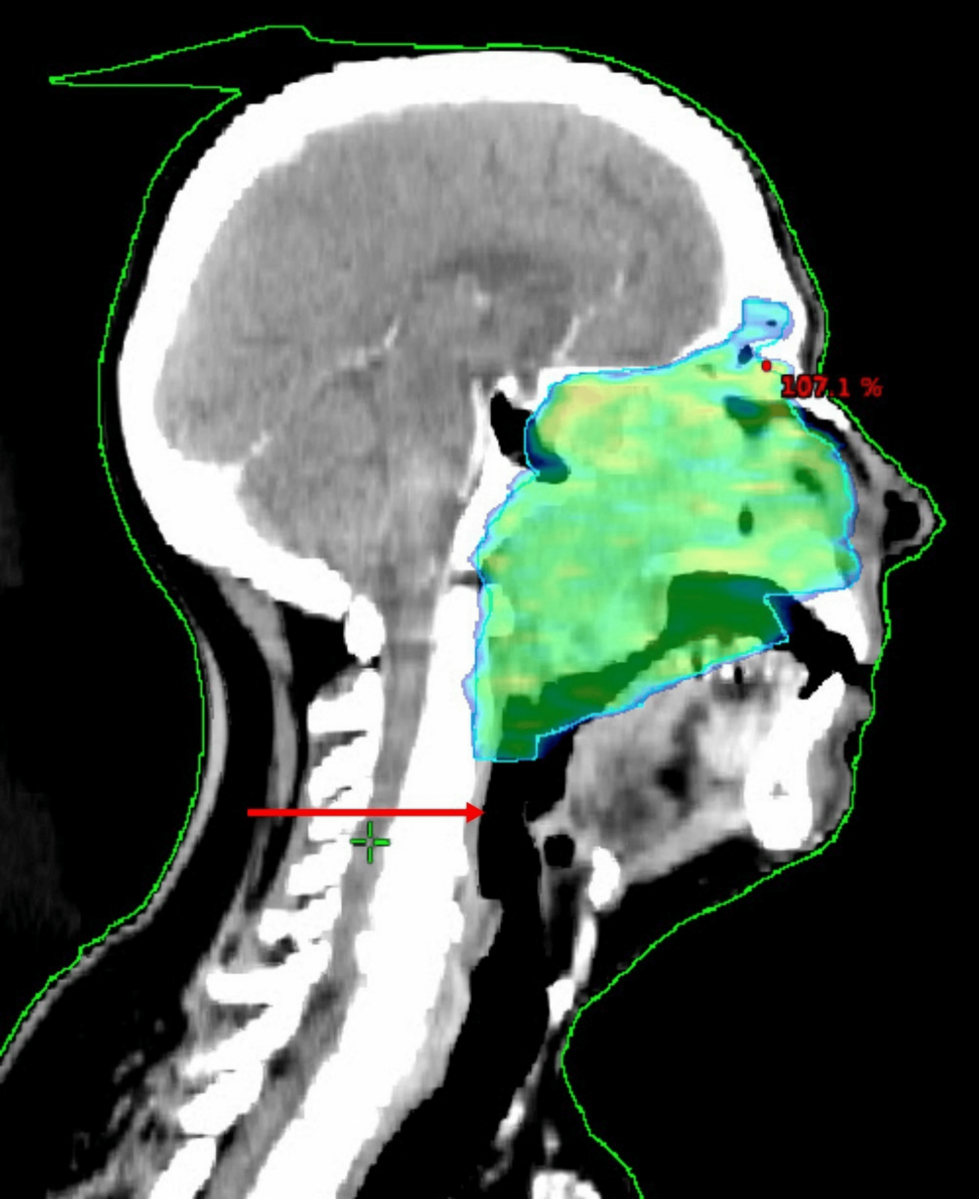
Case 1 with EMP of the nasopharynx treated with adaptive VMAT to a total dose of 55.8 Gy/31# The arrow indicates no elective neck node irradiation was done. EMP: extramedullary plasmacytoma, VMAT: volumetric modulated arc therapy

A 55-year-old male presented to us with nasal obstruction and pain around the nasal area for 15 days. There were no symptoms of visual disturbances. There were no associated comorbidities and no significant family history or genetic disorder. The patient has been evaluated outside with contrast-enhanced computed tomography (CECT) of the paranasal sinus, which showed a soft tissue lesion in the left nasopharyngeal region, causing significant airway compromise. The lesion is located at the soft palate and left masticator space. There was no associated lymphadenopathy. Biopsy from the nasopharyngeal mass was reported as a differential diagnosis of differentiated nasopharyngeal carcinoma or high-grade non-Hodgkin lymphoma. This was a diagnostic challenge as per the histopathology report. Subsequently, the patient underwent positron emission tomography-computed tomography (PET-CT), which showed a large, ill-defined lesion in bilateral ethmoid air cells, maxillary sinus, sphenoid sinus with extension to the nasal cavity, and nasopharynx (SUVmax: 9). Immunohistochemistry (IHC) was requested, which was reported as CD138+ and MUM+. After that, investigations related to plasmacytoma were done. In serum electrophoresis (SEP), no M band was detected, and bone marrow aspiration (BMA) showed infiltration with 5% plasma cells. Blood parameters such as complete blood count (CBC), electrolytes, serum lactate dehydrogenase (LDH), and serum microglobulin were normal in range. An assay on serum light chains was not done. So, a diagnosis of EMP with minimal bone marrow involvement was made. The patient was treated with definitive adaptive radiotherapy by volumetric modulated arc therapy (VMAT) (Figure 1) to a dose of 45 Gy/25#/5#/week followed by a boost dose of 10.8 Gy/6#/5#/week in phase 2 on 6 MV linear accelerator (LINAC). The total dose was 55.8 Gy/31#. Dose to organs at risk (OARs) were well within limits. Planning CT was done again for the boost phase. No elective regional nodal irradiation was given in this case (Figure 2).

The patient tolerated the treatment well. Only grade 1 skin and grade 2 mucosal reactions were observed. Now, the patient has been in complete remission for three years evaluated by PET-CT.

Case 2

A 45-year-old male complained of pain in his right shoulder for three months duration. On examination, there was ill-defined swelling in the right upper arm with restriction of movement observed for all types of movements. On the visual analog scale (VAS), his pain was scored as a 4 (very severe). He was initially evaluated elsewhere and suspected to be a case of giant cell tumor according to plain X-ray findings. However, fine needle aspiration cytology (FNAC) gave a diagnosis of mucocystic carcinoma of the humerus. At our place, he underwent CECT of the right shoulder and arm. The CECT showed a large expansile lytic lesion in the right humerus with erosion of the meta-diaphyseal region along with some soft tissue components infiltrating the medial compartment. The glenohumeral joint space and acromioclavicular joint were normal. High-resolution computed tomography (HRCT) of the thorax revealed no bony or soft tissue or lung lesions. CECT of the abdomen and pelvis reported no obvious bony or soft tissue lesions. A core biopsy from the lesion was done. On microscopy, the tumor composed of neoplastic plasmacytoid cells displaying focal ulceration, and vesicular nuclei and prominent nucleoli were seen. SEP showed no M peak, and no plasma cells were found on BMA. His blood parameters CBC and electrolytes were in the normal range. Therefore, a diagnosis of SBP was made, and the patient was planned for definitive radiotherapy. He received a total dose of 54 Gy/27#/5#/week by three-dimensional conformal radiotherapy (3DCRT) on 6 MV LINAC. He tolerated the treatment well, and only grade 1 skin toxicity was observed. Now, he is in complete clinical remission and pain-free for one year. He has a VAS score of zero post-RT.

Case 3

**Figure 3:**
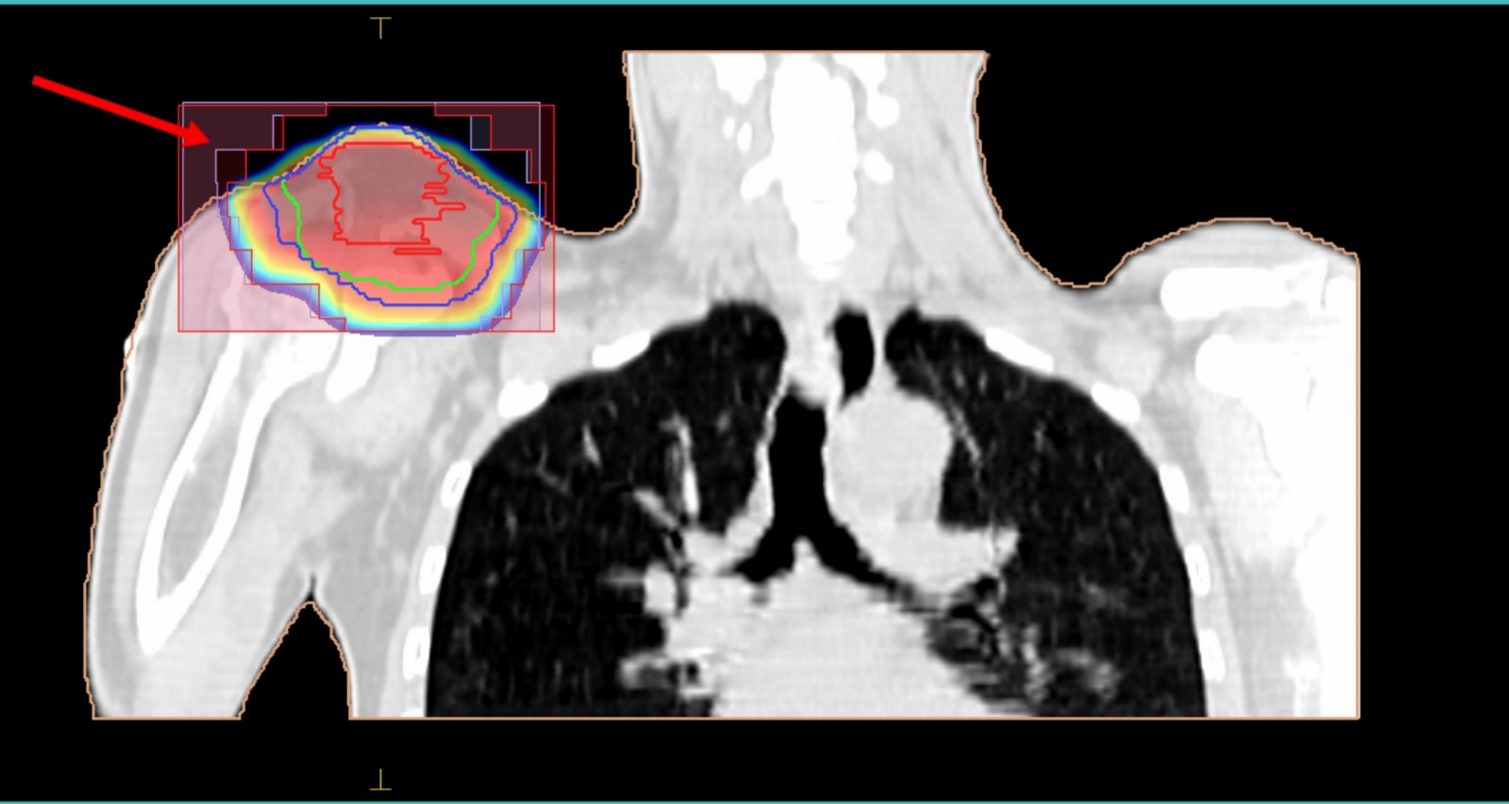
Case 3 with SBP of the right clavicle The arrow points to the target coverage by 3DCRT. The image is in the bone window. SBP: solitary bone plasmacytoma, 3DCRT: three-dimensional conformal radiotherapy

A 52-year-old male with no comorbidities and without any significant family and genetic history presented with right shoulder pain and swelling for three months duration. There was a bony hard swelling at the distal end of the right clavicle measuring 7 × 5.5 cm. His VAS was 4 (severe pain). There was restriction of movement with abduction limited to less than 30 degrees. FNAC from the swelling was suggestive of plasmacytoma. The core biopsy from the lesion was also suggestive of plasmacytoma/myeloma. PET-CT showed fluorodeoxyglucose (FDG)-avid lytic lesion with cortical erosion and associated soft tissue density in the lateral end of the clavicle (SUVmax: 9.89). No M peak was observed on SEP. BMA showed no evidence of plasma cells in bone marrow. So, this patient was treated with 50 Gy/25#/5#/week by 3DCRT (Figure 3).

The patient well tolerated the treatment, and grade 1 skin toxicity was observed during and at the end of the radiotherapy. His pain completely subsided, VAS reached zero, and his shoulder movements improved with an abduction of more than 90 degrees. At three months of follow-up, he will be evaluated with CECT to look for any ossification in the primary lesion, and if feasible, the patient will be advised PET-CT to see the FDG uptake and assess the response.

Case 4

**Figure 4:**
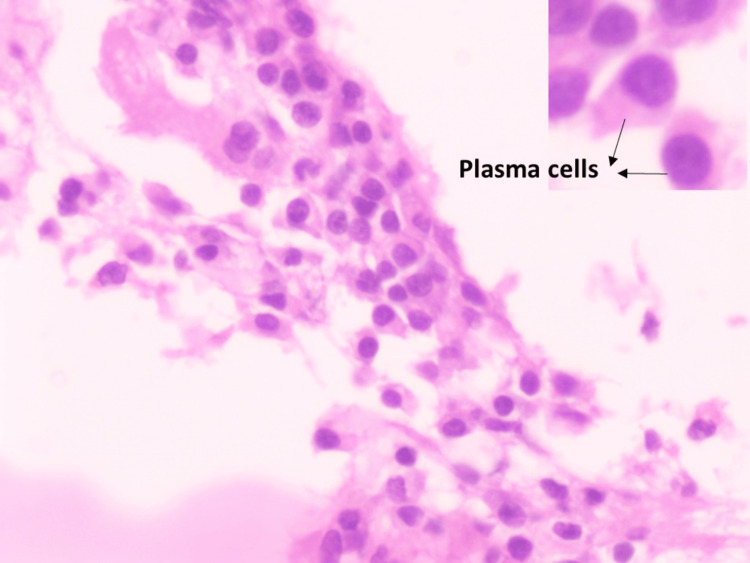
Microphotograph of case 4 showing sheets of plasma cells (H&E stain, 40×) H&E: hematoxylin and eosin

**Figure 5:**
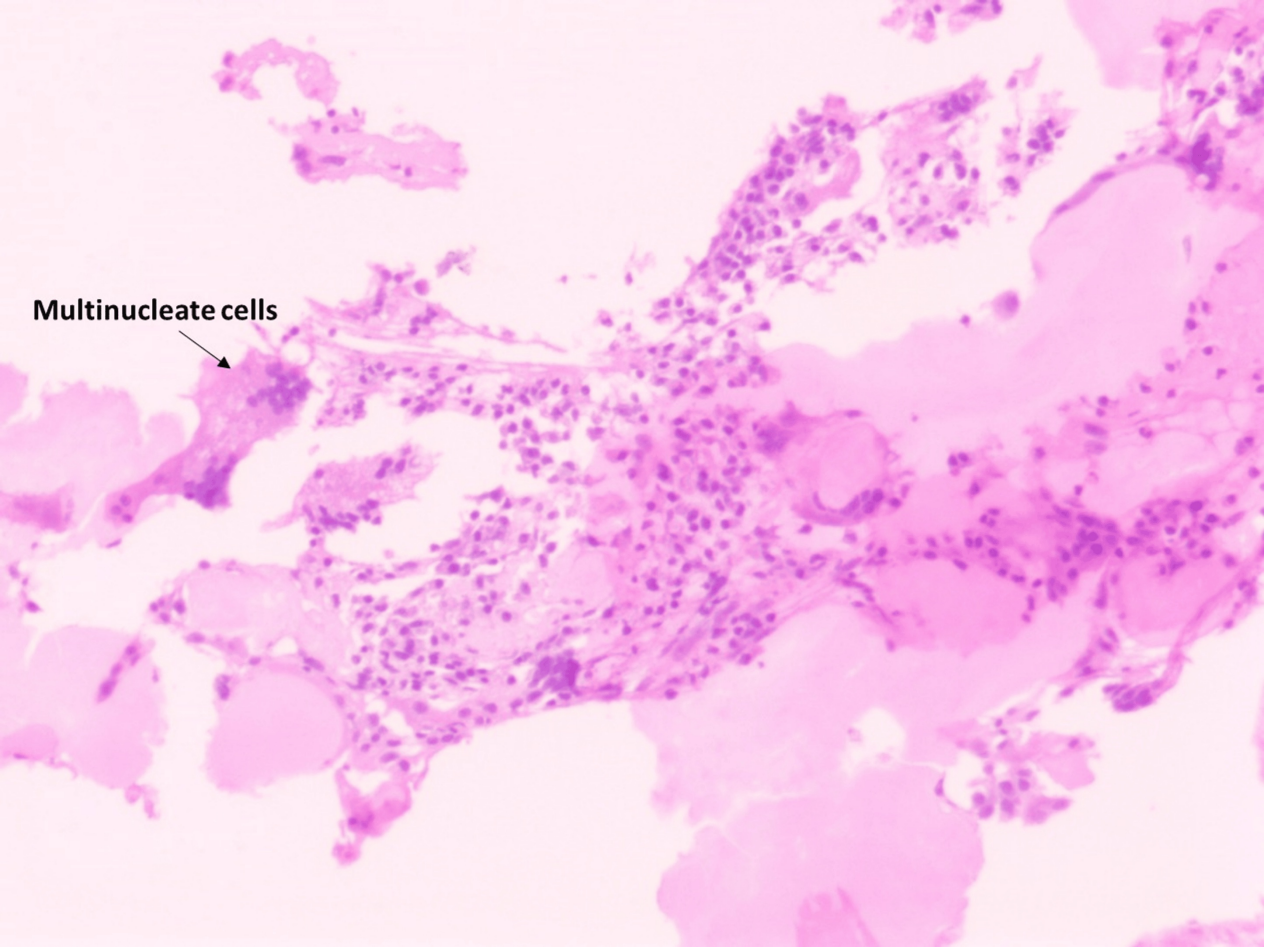
Microphotograph of case 4 showing fibrocollagenous and bony tissue with aggregates of plasma cells, lymphocytes, and histiocytes; few binucleated plasma cells and multinucleated giant cells are seen, as well as areas of necrosis (H&E stain, 10×) H&E: hematoxylin and eosin

A 50-year-old male with complaints of low back ache and inability to walk for one month duration was first seen in the neurosurgery department. He had paraplegia and a VAS of 3 for pain. Magnetic resonance imaging (MRI) of the whole spine demonstrated a focal, well-defined lesion involving epidural space involving D4-D5 vertebral levels causing significant canal stenosis and severe cord compression. HRCT of the thorax and CECT of the abdomen and pelvis showed no evidence of any lesion other than the D4-D5 bony, soft tissue lesions or lymphadenopathy. No M peak was observed in SEP. CBC and electrolytes were normal. The patient underwent a laminectomy. The biopsy report showed neoplastic plasmacytoid cells. Microscopy details are described in Figure 4 and Figure 5.

**Figure 6:**
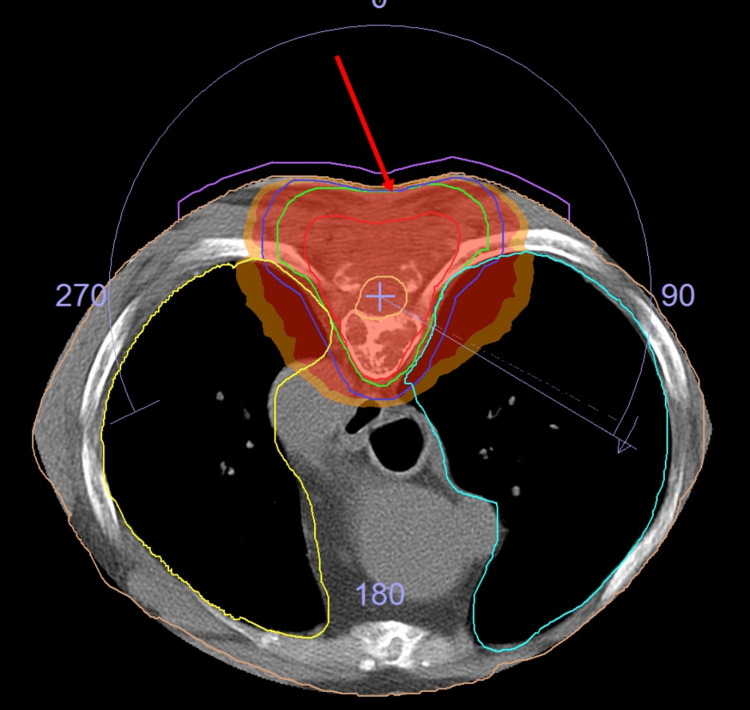
Case 4 with SBP of D3-D4 vertebrae post-laminectomy The arrow points to the target coverage by VMAT planning. D3-D4: dorsal, VMAT: volumetric modulated arc therapy

IHC was positive for CD138 and Kappa but negative for Lambda. Taking morphology and IHC into consideration, a diagnosis of plasmacytoma was confirmed. He was planned for adjuvant radiotherapy by VMAT to a total dose of 40 Gy/20# (Figure 6). At the time of radiotherapy, the patient's attendant refused radiotherapy as they had a misbelief that radiotherapy would lead to the progression of the disease. So, no outcome data is available for this patient.

## Discussion

Plasmacytoma has a natural history of transforming into myeloma, and the rate is 50%-60% over 10 years for SBP and even less for EMP, i.e., 20%-30% [3].

Plasmacytoma with minimal bone marrow involvement (plasma cells < 10%) has been recognized as a prognostic factor for rapid progression to multiple myeloma (MM). Over 2-3 years, 60%-70% of SBP and 20% of EMP patients transform to MM [4].

As solitary plasmacytoma (SP) is characterized by a single lesion in either bone or soft tissue, to rule out the presence of any other bony or soft tissue lesions in the body, the patient should undergo either CECT, MRI, or PET-CT. Conventional skeletal surveys by X-rays are not favored in the present day due to the wider availability of 3D imaging as X-rays have less sensitivity to detect early lesions because a minimum of 30% bone destruction is required for its depiction by X-rays. Nevertheless, X-rays are less sensitive for evaluating the extent of soft tissue lesions in SEP. MRI has superiority over CT for extradural lesions depicting cord compression, neural-foraminal involvement, skull base tumors, and diffuse marrow involvement. FDG PET-CT is mandatory [1] to confirm a suspected case of plasmacytoma as it has superiority over both MRI and CT, and it can detect approximately 30% of lesions missed by these. However, it has a limitation of missing small lytic lesions of the skeleton, especially the skull, and early diffuse bone marrow involvement. So, this newer imaging at times upstages the disease and leads to clinical dilemmas about whether it is plasmacytoma or MM.

All suspected cases of solitary plasmacytoma should be tested for CBC with peripheral blood smear (PBS), serum electrolytes, kidney function test (KFT) along with LDH, beta 2 microglobulins, serum and 24-hour urine electrophoresis to detect M peak, and serum light chain assay to rule out MM, as MM is associated with CRAB (hypercalcemia, renal insufficiency, anemia, and bone lesions) features. BMA/biopsy and IHC are mandatory to make a diagnosis of plasma cell neoplasm. IHC markers include CD138, MUM, Kappa, and Lambda. Occult marrow involvement is to be detected by flow cytometry [5].

Radiotherapy has been the cornerstone in the treatment of SP. Still, there has been no uniform consensus on radiotherapy dosage for SP. Radiotherapy with dosages of 50-60 Gy has been used in studies [6]. However, no dose-response relationship with radiotherapy has been demonstrated. There has been evidence that tumors less than 5 cm respond well to doses up to 35 Gy. However, the National Comprehensive Cancer Network (NCCN) recommends 40-50 Gy for all SP. Higher dosage or hypofractionation schedules have been advocated for SP with minimal bone marrow involvement. Elective nodal irradiation in the absence of nodes as evident on imaging is not recommended in EMP, as chances of failure have been found to be very low (<5%) [7]. In our study, in the first case of EMP, we have gone up to 55.8 Gy/31# by adaptive radiotherapy respecting the constraints of OARs. The basis of this dose prescription was that a dose higher than 55 Gy has been the standard of care in the paranasal sinus/nasopharynx. So, this dose regimen will be safe for the OARs. The patient has achieved three-year disease-free survival (DFS) without any long-term toxicity to date. Similarly, in our second case, we have gone up to 54 Gy/27#. We know that 54-60 Gy is a safe dose for other tumor types such as sarcoma in this axial region. The patient is in clinical remission for one year.

Surgery plays a role in the stabilization of fractures involving long bones and in laminectomy and decompression for extradural disease causing cord compression, It is also used for vertebral stabilization for fractured vertebrae causing impingement over the spinal cord and pain [8]. However, adjuvant RT is often required in these cases; otherwise, high failure rates have been reported. Wide local excision has been achieved only in very small, superficial tumors or tumors in visceral structures such as the lung. In these cases, adjuvant RT may be omitted.

Apart from minimal bone marrow involvement (10%), there are other prognostic factors associated with SP: persistent active metabolic activity on post-treatment FDG PET, tumor size > 5 cm, increased clonal light chains, and persistent M peak in secretory cases of plasmacytomas [9]. These subgroups of patients have a tendency for local failure as well as progression to MM. Adjuvant chemotherapy/novel agents are evolving areas of research for these subsets of patients. However, as the disease is rare, patient recruitment has been a problem for randomized controlled trials (RCTs). A similar issue was addressed in UK-based IDRIS RCT [10], where a target number of patients could not be recruited and premature closure of the study was done. However, results were promising with prolonged progression-free survival (PFS) with lenalidomide and dexamethasone post-RT than RT alone in high-risk cases (occult marrow involvement and abnormal serum-free light chains). Individual patient data has been reported using bortezomib, dexamethasone, and bisphosphonates [11]. Remineralization of bone was achieved in SBP with prolonged remission without radiotherapy. However, overall definitive evidence is lacking in this setting.

Baseline imaging should be done to assess response. However, bony lesions take 6-8 months to respond. Because they remain lytic for an extended period, ossification of the bones takes longer. Here, FDG PET plays a role, where the lesion can become non-avid. Also, secretory type plasmacytoma may have no M peak detectable in completely responding tumors [6].

## Conclusions

Plasmacytoma is a rare disease with varied site presentation either as bony or extramedullary. There are challenges in the diagnosis to get the tissue and to differentiate it from metastatic disease or MM. Radiotherapy is a curative modality in the majority of cases and gives patients a pain-free life. Determination of high-risk category is to be considered to optimize outcomes. However, integration of adjuvant novel therapy is an area of interest and needs to be further explored.

## References

[1] Rajkumar SV, Dimopoulos MA, Palumbo A (2014). International Myeloma Working Group updated criteria for the diagnosis of multiple myeloma. Lancet Oncol.

[2] Gagnier JJ, Kienle G, Altman DG, Moher D, Sox H, Riley D (2014). The CARE guidelines: consensus-based clinical case report guideline development. J Clin Epidemiol.

[3] Kilciksiz S, Karakoyun-Celik O, Agaoglu FY, Haydaroglu A (2012). A review for solitary plasmacytoma of bone and extramedullary plasmacytoma. ScientificWorldJournal.

[4] Paiva B, Chandia M, Vidriales MB (2014). Multiparameter flow cytometry for staging of solitary bone plasmacytoma: new criteria for risk of progression to myeloma. Blood.

[5] Cavo M, Terpos E, Nanni C (2017). Role of 18F-FDG PET/CT in the diagnosis and management of multiple myeloma and other plasma cell disorders: a consensus statement by the international myeloma working group. Lancet Oncol.

[6] (2018). Erratum to: Tsang RW, Campbell BA, Goda JS et al. radiation therapy for solitary plasmacytoma and multiple myeloma: guidelines from the International Lymphoma Radiation Oncology Group. Int J Radiat Oncol Biol Phys 2018; 101: 794-808. Int J Radiat Oncol Biol Phys.

[7] Sasaki R, Yasuda K, Abe E (2012). Multi-institutional analysis of solitary extramedullary plasmacytoma of the head and neck treated with curative radiotherapy. Int J Radiat Oncol Biol Phys.

[8] Soutar R, Lucraft H, Jackson G, Reece A, Bird J, Low E, Samson D (2004). Guidelines on the diagnosis and management of solitary plasmacytoma of bone and solitary extramedullary plasmacytoma. Br J Haematol.

[9] Caers J, Paiva B, Zamagni E (2018). Diagnosis, treatment, and response assessment in solitary plasmacytoma: updated recommendations from a European Expert Panel. J Hematol Oncol.

[10] Kushani E, Rabin NK, D'Sa S (2022). Adjuvant systemic therapy in high risk solitary bone plasmacytoma: results of the UK randomised phase III Idris study (CR UK/14/032). Blood.

[11] Blum A, Bazou D, Ting KR (2021). Successful treatment of solitary bone plasmacytoma and bone remineralisation with novel biological agents leading to new bone formation - a case series. Br J Haematol.

